# Estradiol Inhibits Human Brain Vascular Pericyte Migration Activity: A Functional and Transcriptomic Analysis

**DOI:** 10.3390/cells10092314

**Published:** 2021-09-04

**Authors:** Lisa Kurmann, Michal Okoniewski, Raghvendra K. Dubey

**Affiliations:** 1 Department of Reproductive Endocrinology, University Hospital Zurich, 8952 Schlieren, Switzerland; lisa.kurmann@usz.ch; 2 ID Scientific IT Services, ETH Zurich, 8092 Zurich, Switzerland; michal.okoniewski@id.ethz.ch; 3 Department of Pharmacology & Chemical Biology, University of Pittsburgh, Pittsburgh, PA 15219, USA

**Keywords:** estrogen receptor, MAPK, TNFα, blood–brain barrier, stroke, inflammation, COVID-19

## Abstract

Stroke is the third leading cause of mortality in women and it kills twice as many women as breast cancer. A key role in the pathophysiology of stroke plays the disruption of the blood–brain barrier (BBB) within the neurovascular unit. While estrogen induces vascular protective actions, its influence on stroke remains unclear. Moreover, experiments assessing its impact on endothelial cells to induce barrier integrity are non-conclusive. Since pericytes play an active role in regulating BBB integrity and function, we hypothesize that estradiol may influence BBB by regulating their activity. In this study using human brain vascular pericytes (HBVPs) we investigated the impact of estradiol on key pericyte functions known to influence BBB integrity. HBVPs expressed estrogen receptors (ER-α, ER-β and GPER) and treatment with estradiol (10 nM) inhibited basal cell migration but not proliferation. Since pericyte migration is a hallmark for BBB disruption following injury, infection and inflammation, we investigated the effects of estradiol on TNFα-induced PC migration. Importantly, estradiol prevented TNFα-induced pericyte migration and this effect was mimicked by PPT (ER-α agonist) and DPN (ER-β agonist), but not by G1 (GPR30 agonist). The modulatory effects of estradiol were abrogated by MPP and PHTPP, selective ER-α and ER-β antagonists, respectively, confirming the role of ER-α and ER-β in mediating the anti-migratory actions of estrogen. To delineate the intracellular mechanisms mediating the inhibitory actions of estradiol on PC migration, we investigated the role of AKT and MAPK activation. While estradiol consistently reduced the TNFα-induced MAPK and Akt phosphorylation, only the inhibition of MAPK, but not Akt, significantly abrogated the migratory actions of TNFα. In transendothelial electrical resistance measurements, estradiol induced barrier function (TEER) in human brain microvascular endothelial cells co-cultured with pericytes, but not in HBMECs cultured alone. Importantly, transcriptomics analysis of genes modulated by estradiol in pericytes showed downregulation of genes known to increase cell migration and upregulation of genes known to inhibit cell migration. Taken together, our findings provide the first evidence that estradiol modulates pericyte activity and thereby improves endothelial integrity.

## 1. Introduction

Stroke is a major contributor for mortality and disability in women [1] and a burden often overlooked compared to cancer. The overall risk for developing a stroke is higher in women compared to men [2]. Moreover, a dramatic increase in stroke events is largely observed in women after menopause, whereas the risk in premenopausal women is relatively lower compared to age-matched men [2]. These findings, together with the fact that estradiol levels drop following menopause, have led to the hypothesis that estradiol may be protective and may contribute to the sexual dimorphism in cardiovascular diseases, including stroke. Estrogen plays an important role in regulating dynamic changes in vascular structure and function and, importantly, it protects against vascular remodeling associated with coronary artery disease and endothelial injury [3,4]. Apart from being vaso-protective, estrogen is also known to induce neuroprotective actions by preventing uncontrolled neuroinflammation and excessive ROS production. The blood–brain barrier (BBB) is critical for homeostasis of the central nervous system, and BBB disruption is an important aspect in stroke incidents [5]. A widely accepted hypothesis is that estrogen’s protective function is, in part, attributed to the maintenance of endothelial barrier integrity [6,7,8,9,10]. In in vivo animal studies estradiol has largely been shown to protect against injury and ischemic stroke associated capillary leakage and brain damage [6,10,11]. However, conclusive evidence for estrogen mediated BBB protection is lacking, as both, protective and deleterious actions of estrogen on brain capillary endothelial barrier as well as pro-inflammatory action of the hormone have been observed in vitro [12,13,14,15]. Moreover, increased incidents of stroke were also noticed in postmenopausal women taking estrogen replacement therapy [1]. Hence, the overall effects of estradiol on BBB and the underlying mechanism(s) remain inconclusive and require in-depth investigation.

Proper functioning of a dynamic barrier at the blood-brain interface requires highly regulated mechanisms [16]. Although, capillary endothelial cells were long considered to be responsible for BBB integrity, it is now clear that the formation of the BBB is orchestrated by a complex network of different cell types, which together form the neurovascular unit (NVU). In this context, pericytes (PCs), which are located next to a single layer of capillary endothelial cells (ECs) as well as astrocytes, neurons, glial cells and extracellular matrix components contribute to the functional integrity of the BBB [17,18,19,20]. PCs have gained increasing attention in recent years. As vascular mural cells, PCs share the same basement membrane with ECs. They are thought to stem from the same cell lineage as vascular smooth muscle cells (VSMCs) but exhibit important differences with regard to their location in the vasculature, their morphology and function [21,22,23]. Several studies using viable PC-deficient mouse models have demonstrated that reduced PC coverage leads to vascular leakage and brain edema [17,18], thereby shedding new light on the importance of pericyte’s in promoting endothelial integrity. Moreover, PC loss is a hallmark of many central nervous system disorders like Alzheimer’s disease, stroke and multiple sclerosis, to name a few [23,24,25,26,27]. Importantly, the migration of PCs away from the endothelial layer is a critical manifestation of cerebral ischemia and correlates with barrier leakage and break down after stroke, but also in other pathologies like brain trauma, viral infection, sepsis and diabetic retinopathy [28,29,30,31]. Following injury/insult, release of pro-inflammatory cytokines, such as TNFα, have been shown to be a prominent inducer of PC migration in vitro [32,33]. TNFα is also a well-known mediator of vascular dysfunction, and its up-regulation under inflammatory conditions as well as in the plasma of ischemic stroke patients has been shown repeatedly and even correlates with stroke damage and lesion size [34,35].

Based the observations that: in in vivo studies in animals, estradiol is protective against BBB disruption in various models; in in vitro studies using endothelial cell monolayers as a model for BBB function, estradiol has inconclusive actions with protective, deleterious as well as neutral effects; pericytes presence is essential for a functional BBB; following injury (trauma or stroke etc.) PC migration away from the endothelial lining occurs and is a hallmark for BBB disruption; estradiol inhibits migration of SMCs, which are phenotypically similar to pericytes, we hypothesize that estradiol may mediate its protective actions on BBB by modulating PC activity and inhibiting their migration. Indeed, while estrogen action on ECs and SMCs has been extensively investigated [36], its modulatory role in PCs remains unexplored. Since PCs are phenotypically similar to VSMCs as well as the fact that PC degeneration is associated with aging [37], it is feasible that estradiol might also influence PC function. More specifically, we were interested in investigating the effects of estradiol on growth and migration of PCs, and delineating the underlying mechanisms, including estrogen receptor (ER) -α, -β and GPER, as well as protein kinases MAPK and Akt. Furthermore, using PCs co-cultured with endothelial cells, we assessed whether estradiol improves barrier function. To get an in-depth understanding and shed light on the mechanism(s) that may be triggered by estradiol, we analyzed estradiol-induced changes in mono- and co-cultured PCs using transcriptomic analysis.

## 2. Materials and Methods

### 2.1. Cell Culture

hBVPs: Human Brain Vascular Pericytes (HBVPs, ScienCell, Carlsbad, CA, USA) between the 4th and 10th passage were cultured as described previously [38]. Briefly, hBVPs were grown in flasks coated with Poly-L-Lysine (PLL; 2 µg/cm^2^) under standard tissue culture conditions (37 °C, 5% CO_2_) in pericyte growing media (DMEM/F12 supplemented with antibiotic-antimycotic (AA; 100 μg/mL streptomycin, 100 μg/mL penicillin and 0.025 μg/mL amphotericin B), Glutamax (1×) and 10% FBS). The cells were cultured until sub-confluency and media was changed every two or three days.

hCMEC/D3: The human Cerebral Microvascular Endothelial Cell line (hCMEC/D3) [39] was kindly provided by Dr. Pierre-Olivier Couraud (Institute COCHIN, Paris, France). Cells between the 34th and 39th passage were cultured as described before [38] on rat-tail-collagen-coated (250 μg/mL in 80% EtOH) flasks under standard tissue culture conditions (37 °C, 5% CO_2_) in complete growing media (EC basal media (EndoGRO Basal Medium supplemented with 0.2% EndoGRO-LS Supplement, 5 ng/mL rh EGF, 4 mM L-Glutamine, 0.75 U/mL Heparin Sulfate, 50 μg/mL Ascorbic Acid, 1 ng/mL bFGF, antibiotic-antimycotic (100 μg/mL streptomycin, 100 μg/mL penicillin and 0.025 μg/mL amphotericin B) supplemented with 5% FBS. Media was changed every two or three days and cells were passaged after confluency was reached.

### 2.2. Cell Count

hBVPs were plated in PLL-coated 24-wellplates in PC growing media at a density of 5000 cells/cm^2^ and let to attach for 24 h. Cells were growth arrested o/n in starving media (DMEM/F12 supplemented with AA (100 μg/mL streptomycin, 100 μg/mL penicillin and 0.025 μg/mL amphotericin B), GlutaMAX (1×) and 0.5% sf. FCS) for 3 days. Thereafter cells were washed twice, with HBSS (-Mg^2+^ -Ca^2+^), trypsinized with trypsin (0.5%) and counted with a Coulter Counter (Coulter Z1, Coulter Electronics, Luton, UK). Relative cell number was assessed by normalizing to control.

### 2.3. Migration Studies

Migration of cells was assessed by a scratch-/wound-closure-assay. Cells were plated in PLL-coated 24-wellplates and grown to confluence. Cells were starved o/n in starving media (DMEM/F12 supplemented with AA (100 μg/mL streptomycin, 100 μg/mL penicillin and 0.025 μg/mL amphotericin B), GlutaMAX (1×) and 0.5% (sf.) FCS). If required, cells were pre-treated with antagonists/inhibitors for the assigned period of time, before the monolayers were scratched with a yellow pipette tip. Cells were washed once with HBSS (+Mg^2+^ +Ca^2+^) to remove loose cells, and treatment or vehicle was added. Images of each scratch were taken with an automated Olympus IX81 microscope (Olympus, Volketswil, Switzerland) at 0 h and at 10 h after treatment. Area of wound closure was determined by using the software ImageJ, and relative wound closure was calculated as follows: (area(T0)-area(T10))/area(T0). A schematic representation of the experimental set-up is provided in Figure S1.

### 2.4. Western Blot Analysis

For Western blot analysis, cells were grown in 35 mm dishes and treated as specified. For lysis, cells were washed briefly with ice-cold HBSS (+Mg^2+^ +Ca^2+^) before lysis buffer (containing 20 mM Tris pH 7.5, 1% Triton X- 100, 150 mM NaCl, 1 mM EGTA, 1 mM EDTA, 2.5 mM sodium phosphate, 1 mM β-glycerophosphate, 1 mM sodium vanadate, 0.5 PMSF and 0.2% SDS) was added for 2 min on ice. Cell lysates were collected by scraping and samples were frozen at −80 °C until further processing. Concentration of each sample was determined with the Pierce bicinchoninic acid (BCA) Assay Kit according to the manufacturer’s protocol. Equivalent amounts (10 μg) of protein from whole-cell lysates were separated on 8%, 10% or 12.5% SDS-polyacrylamide gels. As a marker, Protein Marker Enhanced 3-color High Range or Precision Plus Dual Color Standard was used. After transfer to a nitrocellulose membrane by the method of wet electroblotting, the membrane was blocked with 5% milk at RT for 1 h. Incubation with the primary antibody was performed o/n at 4 °C. After washing, the membrane was incubated with the secondary antibody for 1 h at RT and washed again. For detection of proteins with IR Dyes, the Odyssey LI-COR system (LI-COR, Lincoln, NE, USA) was used. For peroxidase-conjugated secondary antibodies, chemiluminescent substrates (Pierce, Rockford, IL, USA) were added according to manufacturer’s instruction. Peroxidase activity was detected by exposing the membranes to XOMAT LS films, which were developed with the CAWOMAT 2000 IR film developer (WIROMA AG, Niederscherli, Switzerland).

### 2.5. Transendothelial Electric Resistance (TEER)

For TEER measurements in real-time, a cellZscope instrument (nanoAnalytics GmbH, Münster, Germany) was used. Permeable transparent PET membrane inserts of 0.4 µm pore size and 24-well format (Falcon 353095) were coated with poly-L-Lysine on the basolateral side and with rat tail collagen (250 μg/mL in 80% EtOH) on the apical side for 1 h at 37 °C and washed twice with sterile H_2_O, before they were incubated for another hour in PC growing media (5% FBS). For the co-culture models, hBVPs were seeded either on the basolateral or on the apical side of the insert at a density of 25,000 cells/cm^2^. After adherence of the PCs was achieved (3 h), hCMEC/D3 were seeded in the apical chamber at a cell density of 100,000 cells/cm^2^ in 150 µL EndoGro growing media. Media in the basolateral chamber consisted of PC growing media (5% FBS). After 3 days, media was changed to 2% sf.FBS and hydrocortisone (1 μg/mL) was added. For measurements, inserts were added to the cellZscope instrument (lower chamber: 1 mL media, upper chamber: 0.5 mL media). Establishment of a proper barrier function was monitored by recording resistance values of the inserts every hour, and treatments were only applied once the cells reached a stable baseline. All measurements were normalized to the values of a coated insert without any cells and results are depicted in % of control or in absolute TEER values in ohm × cm^2^.

### 2.6. Microarray

Microarray samples were prepared as described previously [38]: PCs were seeded alone or with ECs on the opposite side of permeable PET membrane inserts of 0.4 μm pore size and six-well format (Corning Incorporated, Corning, NY, USA, Costar 3450) as described above. After 5 days in culture (2% steroid-free FCS (charcoal-stripped) in presence of hydrocortisone), cells were treated with estradiol (10 nM) or vehicle for 48 h. Cells were then trypsinized, centrifuged and lysed in 300 μL RNA lysis buffer (Zymo Research, Irvine, CA, USA). Samples were frozen at −80 °C until further processing. Total RNA was extracted by using the Quick-RNA MiniPrep Kit (ZymoResearch, Irvine, CA, USA, R1055) according to the manufacture’s protocol with a 5417R Centrifuge (Eppendorf, Hamburg, Germany). RNA integrity was checked by calculating the ratio of absorbance at 260/280 nm (>2.0) and 260/230 nm (>1.8). The samples were frozen at −80 °C. Microarray analysis using Affymetrix Clariom S Assay, human (Applied Biosystems by Thermo Fisher Scientific Inc., Waltham, MA, USA, 902927) was performed as previously described [40]. For transcriptome analysis, fragmented biotin-labeled ds cDNA was hybridized to ClariomTM S arrays (ClariomTM S arrays, human). After staining, arrays were scanned with Affymetrix Gene-Chip Scanner-3000-7G (Applied Biosystems by Thermo Fisher Scientific Inc, Waltham, MA, USA) while quality control analysis was performed using GeneChip Command Console Software (GCC) v5.0. Transcriptome analysis was performed at the transcriptomics core facility at the Center for Molecular Medicine Cologne (CMMC). Differentially regulated genes were determined with the Transcriptome Analysis Console (TAC, Applied Biosystems by Thermo Fisher Scientific Inc, Waltham, MA, USA) after uploading the CEL files, based on a fold change cut-off of +/− 1.5 (Log2 FC +/− 0.59) and FDR *p*-value of 0.05. The microarray data are deposited in the public Gene Expression Omnibus (GEO) database under the accession no. GSE168514 (Available online: www.ncbi.nlm.nih.gov/geo/query/acc.cgi?acc=GSE168514) (accessed on 20 July 2021).

### 2.7. Statistical Analysis

Experiments were performed at least 3 times and data are represented as mean +/− SD unless stated otherwise. Statistical evaluation was performed by using R. If ANOVA assumptions were met, parametric testing was performed with one-way ANOVA and subsequent Tukey’s HSD multiple pairwise comparisons. If either one of the ANOVA assumptions were not met, non-parametric testing was performed with Kruskal–Wallis rank sum test and subsequent pairwise Wilcoxon-test with Benjamini & Hochberg corrections for multiple comparisons.

## 3. Results

### 3.1. Estradiol Inhibits Migration of Pericytes but Has No Impact on Proliferation

When we looked at PC proliferation after estradiol (E2) treatment, no significant effect was observed (Figure S2a). As a positive control, we used fetal bovine serum (FBS) (2% and 5%) and platelet-derived growth factor (PDGF) -BB (20 ng/mL) and all of them significantly increased cell number from 100% to 201% (FBS 2%, *p* < 0.0001), to 444% (FBS 5%, *p* < 0.0001) and to 178% (PDGF, *p* < 0.0001), respectively (Figure S2b). Furthermore, the possibility of opposing effects of the different ERs was tested by using ER-agonists. Neither of the used agonists PPT (ER-α-agonist), DPN (ER-β-agonist) and G-1 (GPER-agonist) significantly changed the proliferation of pericytes (Figure S2c).

**Figure 1:**
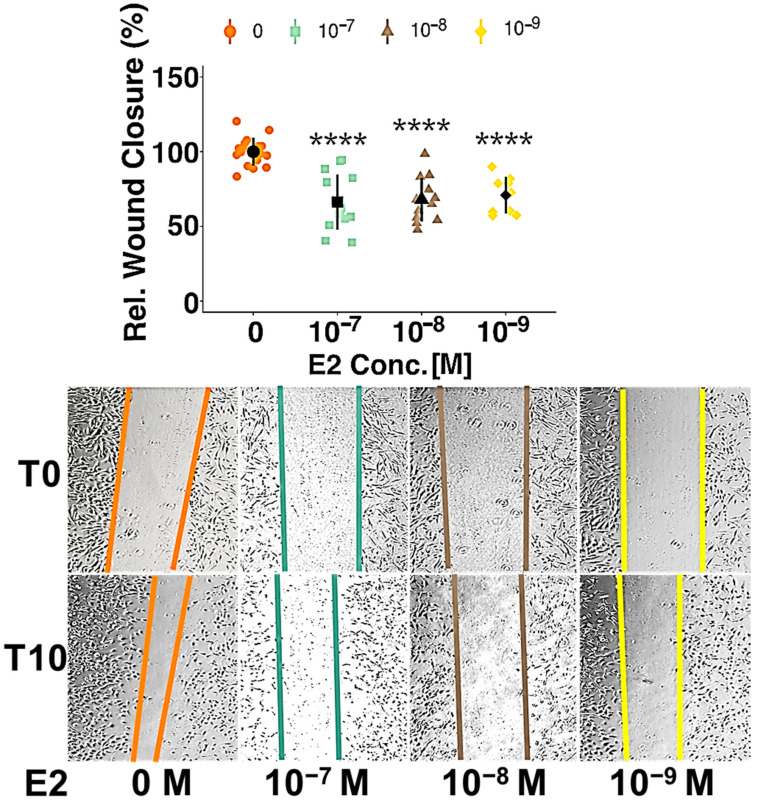
Estradiol inhibits pericyte (PC) migration. Cell migration was assessed using wound healing assays in confluent PC monolayers. Cells were treated with different concentrations of E2 (10^−7^, 10^−8^ and 10^−9^ M) after the scratch was induced and wound closure was determined after 10 h. Representative images are shown for T0 and T10 for each condition Experiments were performed at least 3 times in triplicates and data represent mean +/− sd. **** *p* < 0.0001. “Rel.”: Relative; “Conc.”: Concentration.

To further investigate estradiol’s action on PC function, scratch-wound assays were performed in presence of different concentrations of estradiol. The results showed an inhibition of migration at all considered concentrations of E2 by 30% (10^−7^ M), 32% (10^−8^ M) and 34% (10^−9^ M, all *p* < 0.0001) (Figure 1). PDGF-BB (20 ng/mL) and FBS (2%) were used as positive controls and increased migration by 59% and by 50%, respectively (both *p* < 0.0001) (Figure S3).

### 3.2. Brain Pericytes Express All Three Estrogen Receptors

**Figure 2:**
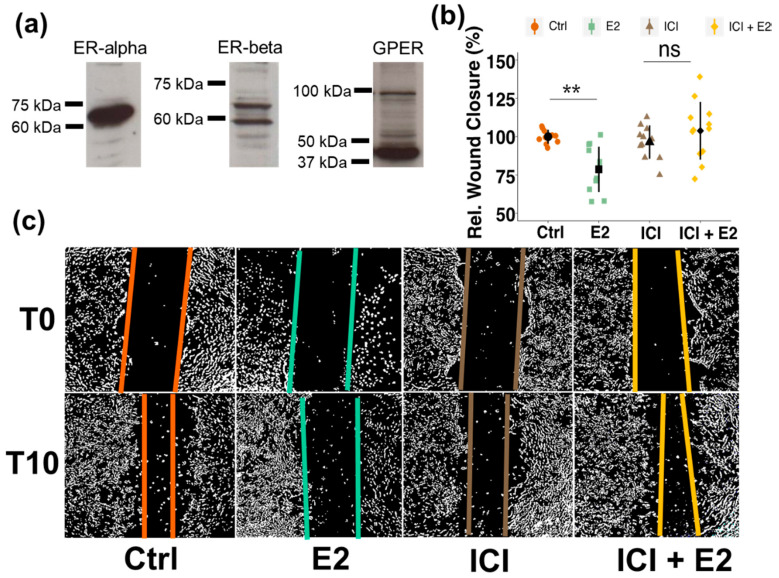
Pericytes (PCs) express all three estrogen receptors (ERs) and mediate inhibitory actions of estradiol (E2) on PC migration. Western blots of PC whole cell lysates showing the presence of ER-alpha, ER-beta and G-protein-coupled ER (GPER) (**a**). Wound healing assays showing the role of ERs in mediating E2 actions on PC migration. The non-selective ER-antagonist ICI 182780 (10^−6^ M) or vehicle was applied for 30 min before making the scratch and cells were treated with E2 (10^−8^ M) or ICI (10^−6^ M) or E2 + ICI and compared to vehicle. Wound closure was assessed after 10 h (**b**). Representative contrast adjusted photomicrographs (for clarity) depicting wounds at T0 and T10 for each condition (**c**). Experiments were performed at least 3 times in triplicates and data represent mean +/− sd. ns *p* > 0.05, ** *p* < 0.01. “Rel.”: Relative.

To assess whether inhibitory effects of estrogen on PC migration were ER mediated, the expression of estrogen receptors (ERs) in pericytes (PCs) was verified by Western blotting. As shown in Figure 2a, all three ERs (ER-alpha, ER-beta and GPER) are well expressed in brain PCs. The estimated molecular weights of the receptors are 66 kDa for ER-alpha, 58 kDa for ER-beta and 42 kDa for the G-protein coupled ER (GPER). Importantly, as shown in the dot plot and representative contrast enhanced photomicrographs, blocking of ERs by pretreating the cells with the non-selective ER-antagonist ICI 182780 completely abrogated the effects of E2 on PC migration (Figure 2b,c).

### 3.3. Estradiol Attenuates Tumor Necrosis Factor Alpha (TNFα)-Induced Migration of PCs

**Figure 3:**
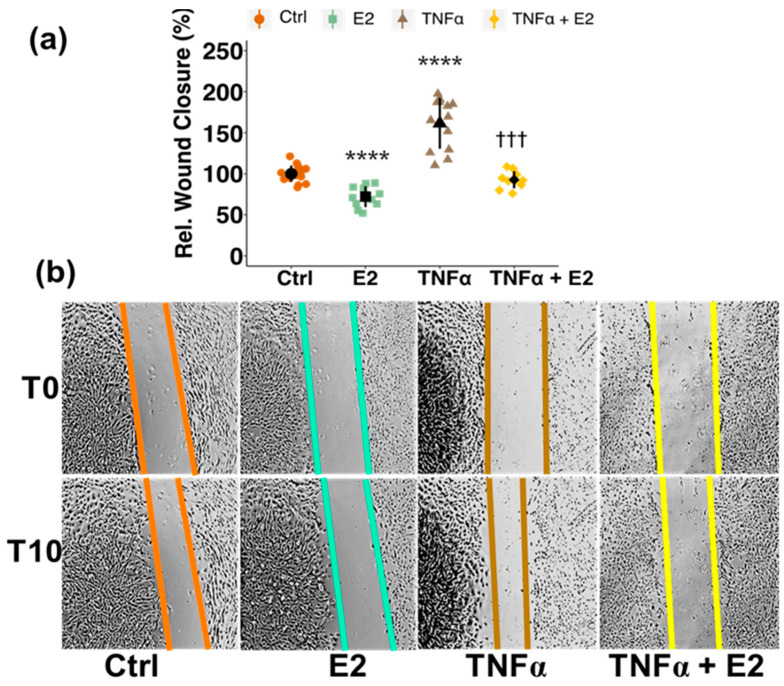
Estradiol (E2) inhibits TNFα-induced pericyte migration. Migration of pericytes was assessed using wound closure assay. After the scratch was made, cells were either treated with E2 (10 nM), TNFα (10 ng/mL), a combination of the two or vehicle (Ctrl) (**a**). Wound closure was assessed after 10 h and representative images are shown at T0 and T10 (**b**). Experiments were performed 3 times in at least triplicates and data represent mean +/− sd. **** p < 0.0001, compared to Ctrl. ††† *p* < 0.001, compared to TNFα. “Rel.”: Relative.

Several studies have shown that following stroke and other brain injuries PCs migrate away from the BBB [30,41,42]. The inflammatory mediator tumor necrosis factor alpha (TNFα) is one of the prominent cytokines known to be upregulated after stroke and is related to BBB break-down, as observed by us and many others [34,43] (Figure S4). Since TNFα has also been shown to increase PC migration in vitro [32,33], we investigated the effect of E2 on TNFα-induced migration of PCs. After confirming the increase in PC migration upon TNFα treatment at several concentrations (0.1 ng/mL–10 ng/mL) (Figure S5), we further applied E2 treatment in combination with TNFα (10 ng/mL). E2 treatment reduced TNFα-induced migration of PCs from 161% (*p* < 0.0001) back to basal levels of 93% (*p* < 0.0001) (Figure 3).

### 3.4. Involvement of Kinase Cascades

**Figure 4:**
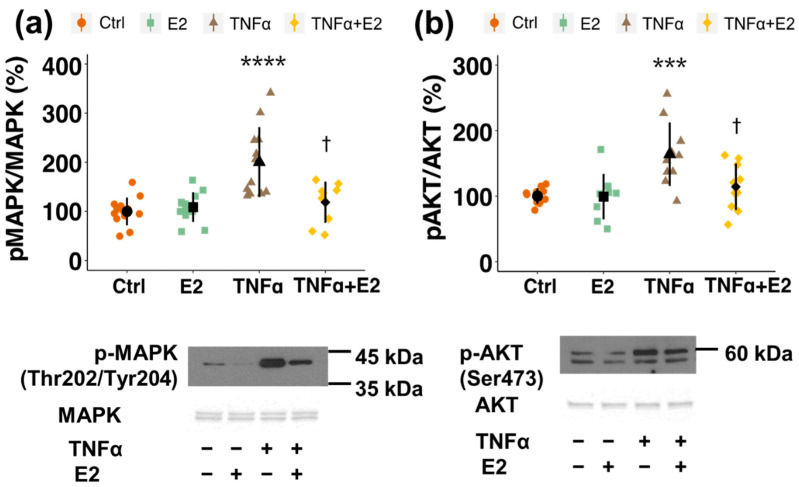
Estradiol (E2) inhibits TNFα-induced phosphorylation of MAPK and AKT kinases. Western blot analysis was performed on whole cell lysates of pericytes treated with E2 (10^−8^ M), TNFα (10 ng/mL), E2 plus TNFα or vehicle (Ctrl) for 6 h. Representative Western blots of MAPK phosphorylation at Threonine residue 202 and Tyrosine residue 204 (**a**) and AKT phosphorylation at Serine residue 473 (**b**) are shown below the graph. Total MAPK and total AKT expression levels were taken for normalization. Experiments were performed 3 times in 3 to 5 replicates each and data represent mean +/− sd. *** *p* < 0.001, **** *p* < 0.0001, compared to Ctrl. † *p* < 0.05 compared to TNFα.

**Figure 5:**
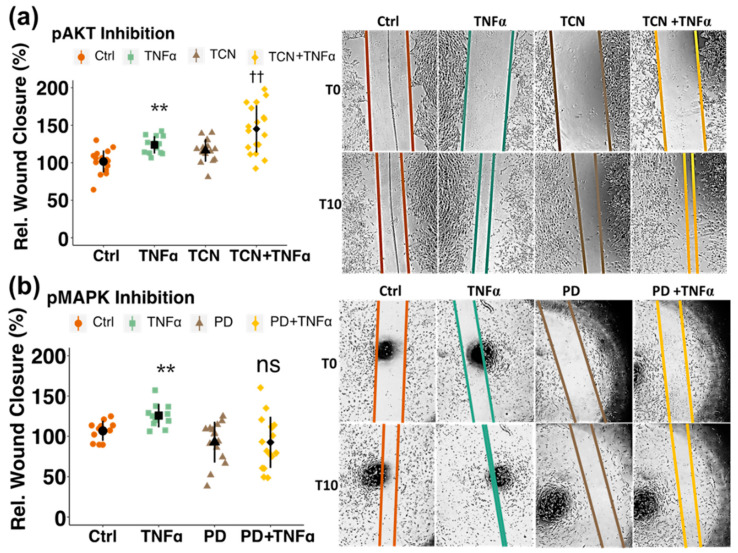
Estradiol (E2) inhibits TNFα-induced pericyte migration by inhibiting pMAPK but not pAKT. Inhibition of pMAPK by pretreating the cells for 30 min with the pharmacological inhibitor PD (10^−5^ M) prevented TNFα-induced increase in migration (**b**), whereas pretreatment with pAKT-inhibitor Triciribine (TCN, 1.5 × 10^−6^ M) showed no effect (**a**). Representative images are shown at T0 and T10 on the right side of the graphs. Experiments were performed 3 times in 3 to 5 replicates each and data represent mean +/− sd. ** *p* < 0.01, compared to Ctrl. ns *p* > 0.05, †† *p* < 0.01, compared to PD or TCN, respectively. “Rel.”: Relative.

**Figure 6:**
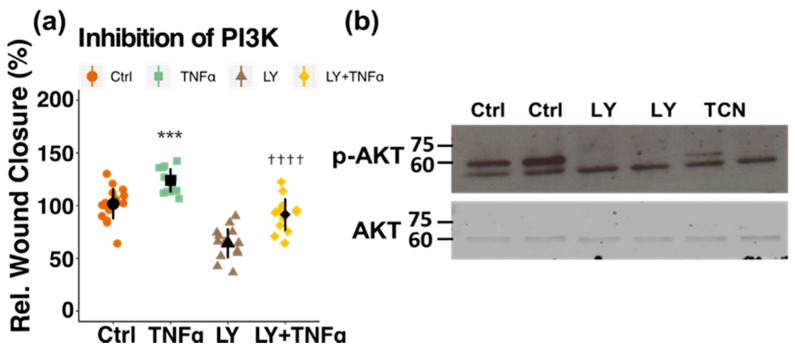
Inhibition of PI3K does not prevent TNFα-induced migration in pericytes (PCs). PCs were pretreated for 30 min with the PI3K inhibitor LY0294002 (LY, 5 × 10^−6^ M) or vehicle, before a scratch was induced and treatment with TNFα (10 ng/mL), LY or a combination of the two was added. The degree of migration was assessed after 10 h (**a**). Experiments were performed three times in 3 to 5 replicates each and data represents mean +/− sd. *** p < 0.001, compared to Ctrl; †††† *p* < 0.0001, compared to LY. “Rel.”: Relative. Treatment with the pharmacological inhibitors for AKT (Triciribine, TCN) and PI3K (LY0294002, LY) resulted in a decrease in AKT phosphorylation at Serine 473 (**b**).

In a next step, the molecular pathway underlying the inhibitory action of estrogen on PC migration was assessed by Western blot analysis and by using pharmacological inhibitors. E2 reduced TNFα-induced phosphorylation of MAPK (at Thr202/Tyr204) from 200% (*p* < 0.0001) back to 119% (*p* < 0.05) and of AKT (at Ser473) from 164% (*p* < 0.001) to 114% (*p* < 0.05) (Figure 4a,b). By treating the cells with pharmacological inhibitors for the two kinases, only pMAPK inhibitor (PD 98059) prevented TNFα-induced migration, whereas pAKT inhibition with triciribine (TCN) showed no inhibitory effect on PC migration (Figure 5a,b). To further confirm this finding, a pharmacological inhibitor for PI3K, which is an upstream kinase of AKT, was used. Pre-treatment with the PI3K-inhibitor LY0294002 also did not inhibit TNFα-stimulated migration (Figure 6a). Importantly, AKT-phosphorylation was decreased by the applied concentrations of both, LY and TCN (Figure 6b).

### 3.5. Involvement of Estrogen Receptors (ERs)

**Figure 7:**
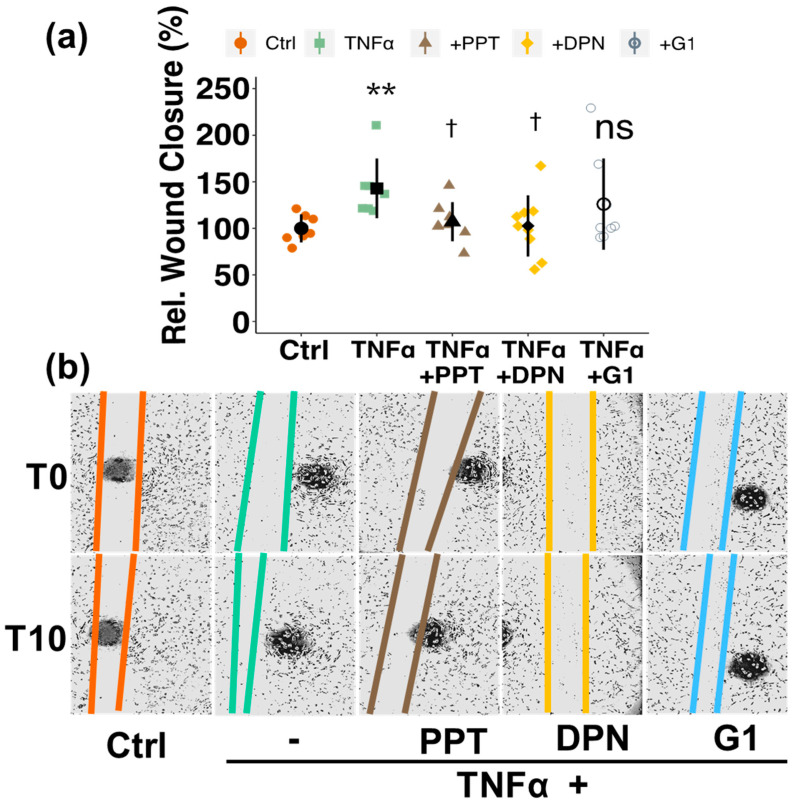
Estrogen receptor (ER)-alpha and ER-beta are responsible for estrogen-mediated downregulation of TNFα-induced migration in pericytes (PCs). PCs were treated with agonists for ER-α (PPT), ER-β (DPN) and GPER (G-1) (10^−7^ M) or vehicle in presence of TNFα (10 ng/mL) after a scratch wound was induced (**a**). Relative wound closure was assessed after 10 h and representative images are shown on the right side of the graph for T0 and T10 (**b**). Experiments were performed 3 times in triplicates or duplicates and data represent mean +/− sd. ** *p* < 0.01, compared to Ctrl; ns *p* > 0.05, † *p* < 0.05, compared to TNFα. “Rel.”: Relative.

To investigate which ER is responsible for the inhibitory effects of E2 on TNFα-induced PC migration, we used selective ER-agonists and -antagonists. ER-α-agonist PPT reduced TNFα-induced migration from 143% to 107% (*p* < 0.05) and ER-β-agonist DPN to 103% (*p* < 0.05), while no significant effect was observed with G-1, the selective agonist for GPER (Figure 7a,b). PPT and DPN mimicked the inhibitory effect of E2 and inhibited basal PC migration by 27% (PPT, *p* < 0.05) and 32% (DPN, *p* < 0.01) (Figure S6). To confirm the importance of ER-α and ER-β in mediating the effects of estradiol on PC migration, we applied selective ER-α- and ER-β-antagonist. Both, MPP (ER-α-antagonist) as well as PHTPP (ER-β-antagonist) abrogated the inhibitory effects of estradiol on PC migration (Figure S7).

### 3.6. Microarray Analysis of Estradiol Treated Pericytes

**Figure 8:**
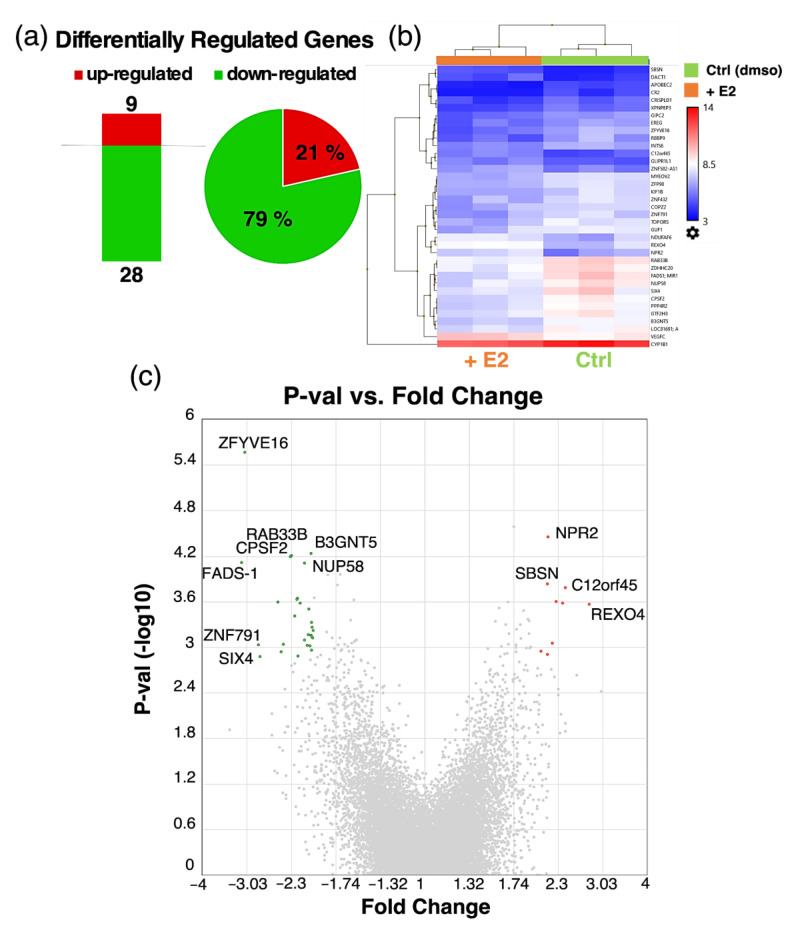
Differentially regulated genes (DRGs) in pericytes (PCs) treated with and without estradiol (E2, 10^−8^ M). Number of DRGs and pie chart representation of up- and downregulated genes in % of total number of DRGs (**a**). Heatmap representation of DRGs between E2- and vehicle (dmso) treated PCs (**b**). Volcano plot showing *p*-value (−log10) on the *y*-axis vs. fold change of DRGs on the *x*-axis. Up- and downregulated genes are depicted in red and green, respectively and the most highly regulated genes are labeled (**c**). Color code: red—upregulated genes; green—downregulated genes. Transcriptome Analysis Console (TAC, Applied Biosystems by Thermo Fisher Scientific Inc., Waltham, MA, USA) was used for analyzing gene expression data of E2 treated vs. dmso treated PCs in triplicates. For the analysis, a fold change (FC) cut-off of 2 (log2FC +/− 1) and FDR *p*-Value of 0.34 was applied.

In order to determine potential genes involved in our observation of decreased cell migration upon estradiol treatment in PCs, we conducted microarray analysis in E2 and vehicle treated PCs. We found 37 differentially regulated genes (DRGs), out of which 9 genes were up- and 28 genes were downregulated upon E2 treatment (Figure 8). A list of the top up- and downregulated genes can be found in the supplementary Tables S1 and S2. Submitting the expression data to pathway analysis yielded no statistically significant results. Interestingly, many of the E2-regulated genes are related to migration as well as metastasis formation in different cancer types (Table 1).

### 3.7. Effect of Estradiol on Endothelial Barrier Function

**Figure 9:**
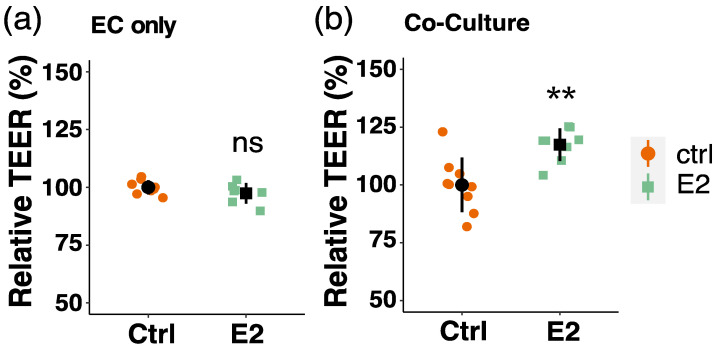
Estradiol increases barrier function in endothelial cells (ECs) in presence of pericytes (PCs). Endothelial cells were grown on permeable transwell inserts alone (**a**) or in co-culture with PCs on the opposite site of the porous membrane (**b**). Cells were treated with estradiol (E2, 10^−8^ M) or vehicle (Ctrl, DMSO) for 48 h and barrier function was measured by means of transendothelial electric resistance measurements with a cellZscope instrument. Experiments were performed 3 times in triplicates or duplicates and data represent mean +/− sd. ns *p* > 0.05, ** p < 0.01, compared to Ctrl.

In order to demonstrate the importance of E2 action on the function of the BBB, we also looked at the effect of E2 by means of barrier function studies with endothelial cells (ECs). We and others previously showed that co-culturing ECs and PCs on the opposite side of a permeable membrane in transwell inserts results in an increased barrier function [40,65,66,67]. Here, we assessed barrier function of ECs by measuring transendothelial electric resistance (TEER) in mono-cultured ECs and ECs co-cultured with PCs in presence or absence of E2. Interestingly, we found that TEER is increased upon E2 treatment in co-cultured ECs (by 17%, *p* < 0.01), whereas no effect of E2 was observed in endothelial monolayers (Figure 9).

### 3.8. Microarray Analysis of Co-Cultured Pericytes Treated with Estradiol

**Figure 10:**
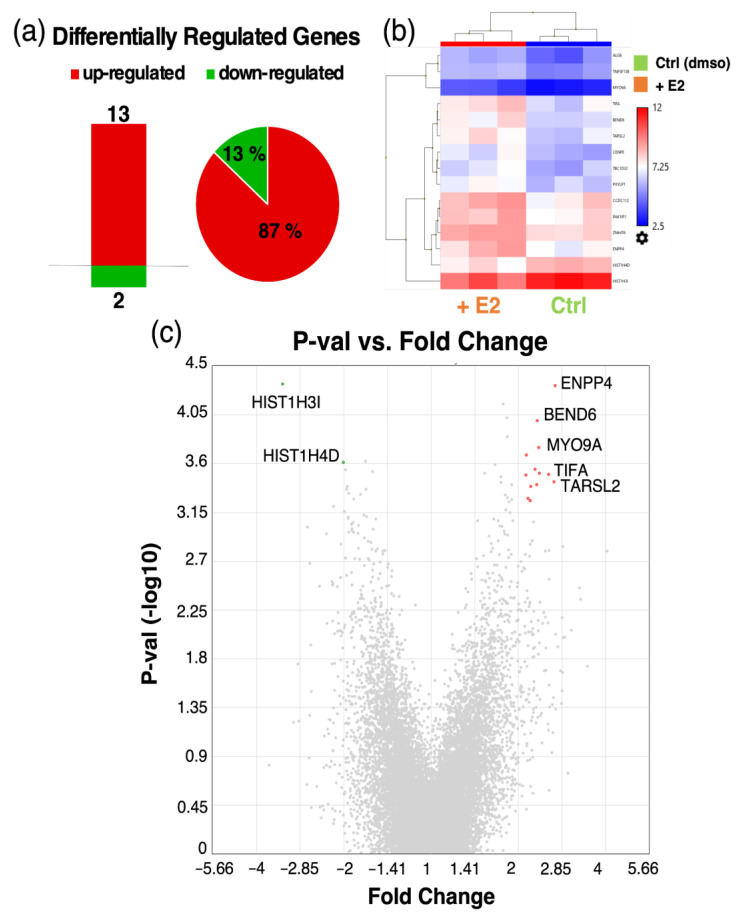
Differentially regulated genes (DRGs) in pericytes (PCs) co-cultured with endothelial cells treated with and without estradiol (E2, 10^−8^ M). Number of DRGs and pie chart representation of up- and downregulated genes in % of total number of DRGs (**a**). Heatmap representation of DRGs between E2- and vehicle (dmso) treated PCs (**b**). Volcano plot showing p-value (−log10) on the *y*-axis vs. fold change of DRGs on the *x*-axis and the most highly regulated genes are labeled (**c**). Color code: red—upregulated genes; green—downregulated genes. Transcriptome Analysis Console (TAC, Applied Biosystems by Thermo Fisher Scientific Inc, Waltham, MA, USA) was used for analyzing gene expression data of E2 treated vs. dmso treated PCs in triplicates. For the analysis, a fold change (FC) cut-off of +/− 2 (log2FC +/− 1) and FDR *p*-Value of 0.34 was applied.

To investigate potential genes and underlying mechanisms that contribute to the observed increase in barrier function (TEER) after E2 treatment in ECs co-cultured with PCs, we performed different microarray analysis in co-cultured cells. While the changes induced by estradiol in mono- and co-cultured endothelial cells were minimal (supplementary Tables S3 and S4), we found a bigger impact of E2 in co-cultured PCs. A total of 15 DRGs was detected, with 13 thereof being up- and 2 being downregulated (Figure 10). The top up-and downregulated genes, when employing a FC +/− cutoff of 2, are listed in supplementary Tables S5 and S6. Interestingly, some of these DRGs are involved in migration/metastasis (TARSL2, BEND6, HIST1H3I, HIST1H4D) as well as barrier function (BEND6) (Table 2).

## 4. Discussion

The protective effects of estrogen on the neurovascular system have extensively been investigated, but mainly with regard to its action on endothelial cells (ECs) and vascular smooth muscle cells (VSMCs), while modulatory effects on pericytes (PCs) were not taken into account. Blood–brain barrier (BBB) disruption is a prominent pathological feature of many different neurovascular disorders, including stroke [5,74]. Since conflicting results regarding effects of estrogen on endothelial barrier integrity have been noticed in the past [6,10,12,13], we hypothesized that PCs might contribute to estrogen’s neuroprotective effects observed in many in vivo as well as clinical and observational studies. The present work demonstrates a novel potential mechanism of estrogen’s neuroprotective action by regulating PC migration.

The expression of estrogen receptors (ERs) has been investigated and confirmed in various regions of the brain in different cell types like ECs, neurons, glial cells as well as astrocytes [75,76,77,78]. However, to the best of our knowledge, their expression in PCs remains unknown. We showed for the first time the expression of all three ERs (ER-α, ER-β and GPER) in human brain PCs on the protein level. Additionally, during our search we found no publications regarding the direct effects of estrogen on PC function, thereby making the findings of the present study unique.

In order to investigate the effects of estrogen action on PC function, we assessed cell proliferation and migration after E2 treatment. E2 is the most potent of the three main estrogens estrone (E1), estradiol (E2) and estriol (E3) [79] and most often used when investigating effects of estrogens in vitro. In PCs treated with E2, we did not observe any effects on cell growth. As positive controls, we used fetal bovine serum (FBS) and PDGF-B, which are common inducers of cell growth in PCs [80]. Both, FBS and PDGF-B induced the growth of PCs. The possibility of a masking effect due to opposing effects of different ERs was also tested by using selective ER-agonists and can be excluded.

Many in vivo and in vitro studies have shown PC degeneration and migration away from the vascular wall acutely after traumatic brain injury, stroke and cerebral ischemia which is correlated with increased permeability of the vessels [30,42,81,82]. Our finding of reduced PC migration upon E2 treatment proposes a novel mechanism by which estrogen might inhibit this important step in disease progression. To highlight our finding also under pathological situations, we induced PC migration by the inflammatory cytokine tumor necrosis factor alpha (TNFα), which is known to be upregulated in several diseases and associated with BBB breakdown in conditions such as ischemic stroke, meningitis and sepsis [83,84,85,86,87]. We have observed its disruptive effects on EC barrier function in our 2D BBB model with the human brain endothelial cell line hCMEC/D3 on porous transwell inserts, confirming what has been shown in other in vitro studies [88,89]. As early as 3 h after ischemic stroke, TNFα mRNA levels are increased and protein expression follows to rise at around 6 h post stroke [90]. Interestingly, we found that E2 stably inhibited TNFα-induced migration of PCs. While the stimulatory effect of TNFα on PC migration has been shown before [91], the inhibitory role of E2 is a novel finding. The counteraction of estrogen and TNFα signaling in the cardiovascular system has been investigated in several in vitro and in vivo studies [92,93,94,95], serving as support for our observation. For example, E2 inhibits TNFα-induced migration and proliferation of SMCs, which are phenotypically similar to PCs [21]. Moreover, different studies demonstrate downregulated TNFα gene transcription and protein expression by estrogens [95,96,97]. The fact that among cytokines, TNFα is one of the most potent inducer of inflammatory responses in PCs [86] highlights the importance of the observed antagonizing action of E2 on TNFα-mediated changes in PC migration.

The molecular mechanism by which E2 inhibits TNFα-induced migration involves repression of MAPK phosphorylation. Despite the fact that E2 reduced TNFα-stimulated phosphorylation of AKT in PCs, inhibiting AKT phosphorylation with pharmacological inhibitors (triciribine and LY0294002) did not mimic the inhibitory effect of E2 on TNFα-stimulated migration in PCs. The applied concentrations of the two inhibitors, however, drastically decreased AKT phosphorylation, demonstrating their inhibitory potency. In contrast to Akt inhibitors, PD98059, the pharmacological inhibitor for ERK1/2, significantly reduced TNFα-induced migration back to basal levels. Increased MAPK-phosphorylation has also been shown in another in vitro study to induce migration in PCs [98]. Furthermore, Takata et al. showed that TNFα induces PC migration via MMP-9 release, and that MMP-9 expression is increased via activation of the PI3K/AKT cascade and MAPK pathways [33]. However, no direct link between AKT-/MAPK-phosphorylation and induced migration was made in this study. Moreover, in rats, the neuroprotective effects of estradiol were linked to attenuated ERK1/2 activation in the ischemic brain [99]. While estrogen signaling is associated with increased phosphorylation of MAPK and AKT in endothelial cells, thereby promoting migration and proliferation, its action on the same kinase cascades in VSMC is just the opposite, with a resulting decrease in mitogenic function. Since PCs are phenotypically similar to VSMCs and seem to stem from the same cell lineage [21], our results fall in line with the previously mentioned observation of a decreased activity of these kinase-cascades upon E2 treatment in VSMCs.

Determining the involvement of ER subtypes is an important step for therapeutics development, since considerable differences exist with regard to function between the three subtypes and ER specificity in different tissues [100,101]. We therefore elucidated the involvement of ERs in mediating estrogen’s inhibitory effects on PC migration and found ER-β as well as ER-α to be responsible for the inhibition of TNFα-induced PC migration by E2. Interestingly, ER-β signaling is responsible for counteracting many other TNFα-induced cellular changes by estrogen in vascular cells [6,93,102,103,104]. Nevertheless, we also observed participation of ER-α in suppressing the TNFα-induced migration in PCs. There are, indeed, different studies showing that the neuroprotective effects of estrogen are mediated by ER-α or a combination of ER-α and ER-β [105,106]. This indicates that there might be different molecular mechanisms by which E2 exerts its anti-inflammatory effects in PCs.

The relevance of PCs with regard to BBB induction and/or protection by estrogen, is further demonstrated by our findings in EC barrier function studies. E2 improved barrier function in EC-PC co-cultures but had no effect on EC monolayers. While an investigation to the underlying cause of this observation was not in the scope of the present study, reduced migration of PCs might be one potential mechanism. Other possible explanations include reduced PC apoptosis, PC contraction or the regulation of signaling molecules between ECs and PCs by E2. Indeed, Glinskii et al. demonstrated effects of E2 on PDGF-B release by ECs which in turn impacts PC recruitment and vessel stabilization. To further gain insights into the intracellular mechanisms that may be involved, transcriptomics analysis was conducted on genes modulated by estradiol in PCs using microarrays. Interestingly in the list of highly regulated genes by estradiol, many were regulators of cell migration. Importantly, estradiol downregulated genes that are known to induce cell migration and upregulated those mediating inhibitory actions on migratory activity. In this context, estradiol down regulated: ZFYVE16, a FYVE zink finger family protein (also called endofin) that regulates cell adhesion and induces cell migration [46] by regulating TGFβ signaling pathway and is downregulated by ER-β in cancer cells [47]; and epiregulin, which stimulates cell migration via MAPK activation [57]. Additionally, estradiol downregulated multiple other genes known to induce cell migration, i.e., B3GNT5, a sphingolipid metabolic enzyme [58]; FADS1 [44], MIR1908 (a cholesterol responsive miRNA [45]); nucleoporin 58 kDa (NUP58) [55]; TOPORS [53,54]; Six homeobox 4 [48]; Retinoblastoma binding protein-9 (RBBP9) [49].

Apart from downregulating genes that induce cell migration, treatment with estradiol upregulated some genes that are involved in cell survival and some are known to inhibit cell migration. In this context, estradiol upregulated: REXO4 or Xenopus gene which prevents mitotic catastrophe (XPMC2) and its human analog, hPMC2, regulate quinone reductase activity via ER-β [59]. Activation of Quinone reductase regulates cell fate decisions during stress/oxidative injury. Importantly, knock down of NAD [P]:quinone oxidoreductase-1 aggravates cancer growth and cell migration [60]; NDUFAF6 or NADH dehydrogenase (ubiquinone) complex 1, assembly factor 6 which is the largest complex of the mitochondrial electron transport chain and known to inhibit cell migration and metastasis [61]. Other tumor suppressor genes which inhibit cell migration, metastasis, invasion and that were induced by estradiol in pericytes include GLIPR1L1 [64]; ZNF582-AS1 (a novel lncRNA) [62]; DACT1 [63]. Additionally, estradiol upregulated: suprabasin (SBSN), a cell differentiation protein, knockdown of which has contractile effects on blood vessels [107], an effect associated with barrier disruption; NPR2 which mediates the neuroprotective actions of C-type natriuretic peptide against hypoxia-ischemia brain injury and regulates microcirculatory flow and blood pressure by acting on pericytes [108]; and VEGF-C, a VEGF isoform which effects lymphatic vessels. VEGF also promotes pericyte growth and coverage of brain capillaries [109]. Together these observations suggest that estradiol largely induces anti-migratory mechanisms to protect and prevent barrier disruption, moreover, it upregulates mechanisms to maintain pericyte survival and well-being.

Although estradiol modulated multiple genes in PC monocultures, it significantly modulated fewer genes in pericytes co-cultured with endothelial cells. Within the upregulated genes TARSL2 [68] and BEND6 [69] have been shown to directly or indirectly inhibit migration/metastasis. Additionally, downregulation of BEND6, a nuclear antagonist of NOTCH, has been observed following BBB disruption [70]. Interestingly, two histone associated genes HIST1H31 and HIST1H4D were downregulated and histone variants are known to facilitate cell motility/migration [71,72,73]. Taken together, our findings from the co-cultured system provide evidence that estradiol may induce its protective action against BBB disruption by preventing the migration of pericytes from endothelial basolateral surface and by increasing factors that promote BBB induction.

A limitation of this study is the pure in vitro character of the performed experiments. Since cell responses can substantially be affected by neighboring cell types, it is essential to verify the present findings also under in vivo conditions. Furthermore, the PCs used for conducting the experiments of this study were of male origin. Since genes expressed on sex chromosomes can have marked impacts on the function of cells, it would be important to perform these experiments also in female cells, in order to make conclusive statements [110]. Furthermore, since only two aspects of PC function have been addressed in this study, it would also be important to investigate the effect of estrogen on other aspects of cellular function such as cell viability under for example hypoxic conditions or in response to TNFα stimulation. Since E2 is a well-recognized inducer of nitric oxide (NO) in the vasculature, and TNFα is associated with reduced endothelial nitric oxide synthase expression and a related decrease in vasorelaxation [97], potential modulatory effects of E2 on ischemia-induced changes in NO production in PCs would be an interesting approach to investigate additional mechanisms of estrogen-mediated neuroprotection.

In two recent publications [38,40], we have shown the modulatory effects of brain microvascular ECs and PCs on genes and BBB function. We also observed dramatic changes in interferon-associated genes and pro- as well as anti-inflammatory cytokines. Collectively, these changes resulted in improved BBB function. Since many of the altered genes were antiviral, together with the fact that capillary pericytes are damaged [111] and interferon levels are decreased in subjects with COVID [112], it is tempting to speculate that the estradiol may protect pericytes against TNFα-induced damage and limit organ damage by preserving barrier function. This may potentially explain the gender based differences in COVID-induced organ damage, disease severity and mortality, where age-matched premenopausal women are protected compared to men, while this effect seems to be lost in postmenopausal women (unpublished findings, doi:10.1101/2020.07.30.20164921) [113]. It is feasible that estradiol may prevent capillary damage by upregulating antiviral mechanisms and preserving pericyte function. This contention is further supported by the fact that TNFα is significantly increased in COVID 19 subjects [114] and our findings that estradiol inhibits TNFα-induced migration of pericytes.

## 5. Conclusions

In conclusion, the present study contributes to a better mechanistic understanding of estrogen’s well-known anti-inflammatory and neuroprotective actions. Our findings postulate that estrogen receptor alpha and -beta signaling might lead to reduced PC detachment from the vessel wall in response to inflammatory stimuli by downregulation of cytokine-induced MAPK phosphorylation, thereby protecting BBB integrity and preventing a cascade of events leading to further aggravation of the condition.

## Figures and Tables

**Table 1:** Differentially regulated genes in estradiol treated pericytes involved in migration and/or metastasis formation.

Gene	Gene Description	Log2 FC (Co- vs. Mono-Culture)	FDR *p*-Value
FADS1 [44]; MIR1908 [45]	Fatty acid desaturase 1; microRNA 1908	−3.1	0.208
ZFYVE16 [46,47]	Zinc finger, FYVE domain containing 16	−3.1	0.058
SIX4 [48]	SIX homeobox 4	−2.8	0.333
RBBP9 [49]	Retinoblastoma binding protein 9	−2.5	0.315
CPSF2 [50]	Cleavage and polyadenylation specific factor 2	−2.3	0.208
RAB33B [51]	RAB33B, member RAS oncogene family	−2.3	0.208
GUF1 [52]	GUF1 homolog, GTPase	−2.2	0.333
TOPORS [53,54]	Topoisomerase I binding, arginine/serine-rich, E3 ubiquitin protein ligase	−2.2	0.249
NUP58 [55]	Nucleoporin 58kDa	−2.1	0.208
PPP4R2 [56]	Protein phosphatase 4, regulatory subunit 2	−2.1	0.315
EREG [57]	Epiregulin	−2.1	0.315
B3GNT5 [58]	UDP-GlcNAc:betaGal beta-1,3-N-acetylglucosaminyltransferase 5	−2.0	0.208
REXO4 [59,60]	REX4 homolog, 3’-5’ exonuclease	2.8	0.249
NDUFAF6 [61]	NADH dehydrogenase (ubiquinone) complex I, assembly factor 6	2.4	0.249
ZNF582-AS1 [62]	ZNF582 antisense RNA 1 (head to head)	2.2	0.315
DACT1 [63]	Dishevelled Binding Antagonist Of Beta Catenin 1	2.1	0.332
*GLIPR1L1* [64]	GLI pathogenesis-related 1 like 1	2.1	0.315

Fold changes (FC) and adjusted *p*-values (FDR *p*-value) are depicted in the third and fourth column, respectively. Transcriptome Analysis Console (TAC, Applied Biosystems) was used for analyzing gene expression data of E2 treated vs. dmso treated PCs in triplicates. For the analysis, a fold change (FC) cut-off of +/− 2 and FDR *p*-Value of 0.34 was applied.

**Table 2:** Differentially regulated genes involved in migration and/or barrier function in estradiol- and control-treated pericytes that were co-cultured with endothelial cells.

Gene	Gene Description	Log2 FC (Co- vs. Mono-Culture)	FDR *p*-Value
TARSL2 [68]	Threonyl-tRNA synthetase-like 2	2.7	0.335
BEND6 [69,70]	BEN domain containing 6	2.3	0.335
HIST1H3I [71,72,73]	Histone cluster 1, H3i	−3.3	0.335
HIST1H4D [71,72,73]	Histone cluster 1, H4d	−2.0	0.335

Fold changes (FC) and adjusted *p*-values (FDR *p*-value) are depicted in the third and fourth column, respectively. Transcriptome Analysis Console (TAC, Applied Biosystems) was used for analyzing gene expression data of E2 treated vs. DMSO treated PCs in triplicates. For the analysis, a fold change (FC) cut-off of +/− 2 and FDR *p*-Value of 0.34 was applied.

## Data Availability

All data supporting the findings of this study are available within the article and its supplemental data file or from the corresponding author upon reasonable request.

## Supplementary Materials

The following are available online at https://www.mdpi.com/article/10.3390/cells10092314/s1. Figure S1. Schematic representation of experimental set-up for the scratch/wound-closure assay. Figure S2. Estradiol (E2) has no impact on pericyte (PC) proliferation/platelet-derived growth factor (PDGF)-BB and fetal bovine serum (FBS) increase PC proliferation/ER-agonists have no effect on PC proliferation. Figure S3. FBS and PDGF-BB induce migration in pericytes (PCs). Figure S4. TNFα reduces barrier function of an endothelial monolayer in vitro. Figure S5. TNFα induces pericyte (PC) migration. Figure S6. Estrogen receptors ER-α and ER-β are responsible for estrogen-mediated downregulation of pericyte migration. Figure S7. ER-α- and ER-β-antagonists block the effect of estradiol (E2) on pericyte (PC) migration. Table S1. Top ten downregulated genes in estradiol treated pericytes; Table S2. Upregulated genes in estradiol treated pericytes; Table S3. Differentially regulated genes (DRGs) in estradiol treated endothelial cells; Table S4. Differentially regulated gene (DRG) in endothelial cells (ECs) co-cultured with pericytes (PCs) and treated with estradiol; Table S5. Downregulated genes in estradiol treated pericytes co-cultured with endothelial cells on the opposite side of a transwell insert; Table S6. Top ten upregulated genes in estradiol treated pericytes co-cultured with endothelial cells on the opposite side of a transwell insert.
