# Supplementary material for: Estradiol Inhibits Human Brain Vascular Pericyte Migration Activity: A Functional and Transcriptomic Analysis
Source: Cells. 2021 Sep 4;10(9):2314. doi: 10.3390/cells10092314 (PMC8472363; doi:10.3390/cells10092314)
Supplement: Supplementary file 1 [file cells-10-02314-s001.zip › cells-1337002-supplementary.pdf]

cells MDPI

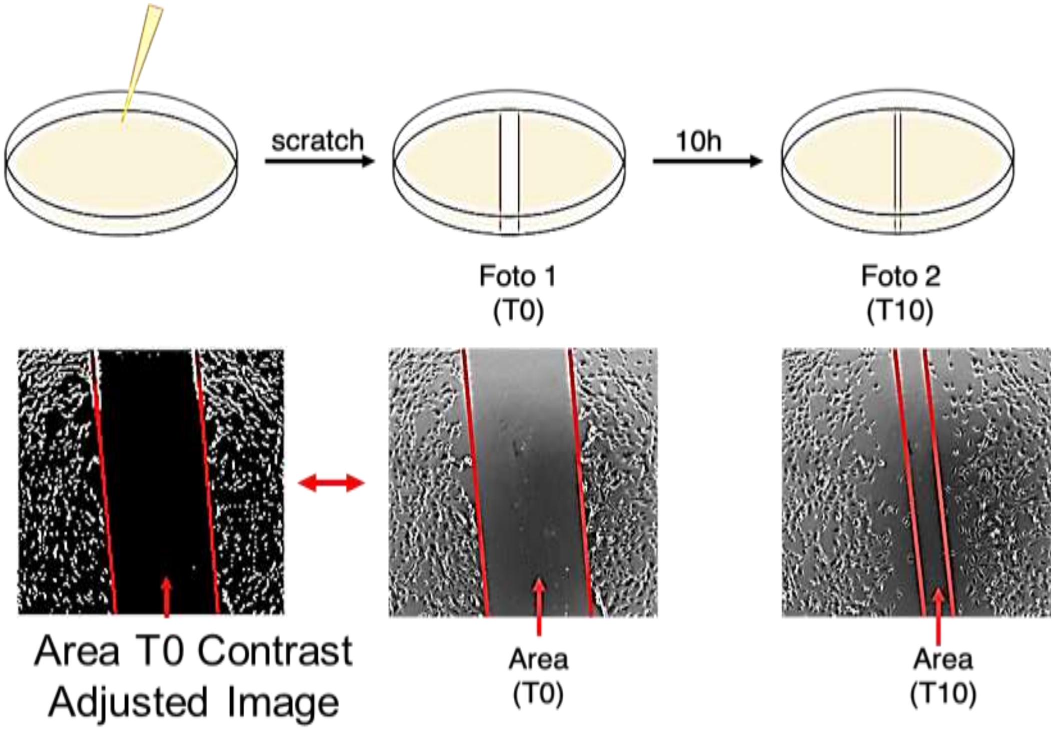

Figure S1. Schematic representation of experimental set-up for the scratch/wound-closure assay. Confluent monolayers were mechanically scratched with a yellow pipette tip (10 µl–200 µl) before treatment was applied. Images of each scratch were taken right after the scratch was made (T0) and 10h thereafter (T10) with an automated Olympus IX81 microscope (Olympus, Volketswil, CH). Area of wound closure was determined by using the software ImageJ, and relative wound closure was calculated as follows: Area(T0)-Area(T10)) / Area(T0). Representative images are depicted.

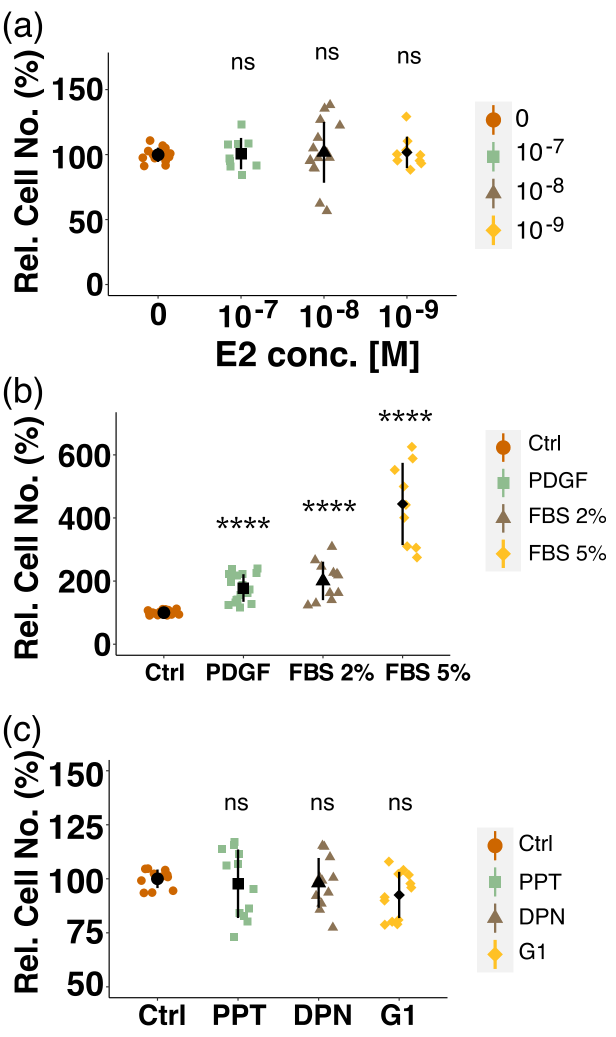

Figure S2. Estradiol (E2) has no impact on pericyte (PC) proliferation / platelet-derived growth factor (PDGF)-BB and fetal bovine serum (FBS) increase PC proliferation / ER-agonists have no effect on PC proliferation. PCs were treated with E2 at different concentrations (10^{-7}, 10^{-8} or 10^{-9} M) or vehicle (**a**) or with PDGF-BB (20 ng/ml) or FBS (2% and 5%) or vehicle (Ctrl) (**b**) or with the ER-agonists PPT (ER-α-agonist), DPN (ER-β-agonist), G-1 (GPER-agonist) (10^{-7} M each) or vehicle **(c)**. Cell number was assessed after 3 days of treatment by cell counting. Experiments were performed at least 3 times in triplicates and data represent mean +/- sd. ns $p > 0.05$, **** $p < 0.0001$ compared to Ctrl. “Conc.” = Concentration; “Rel. Cell No.” = Relative cell number.

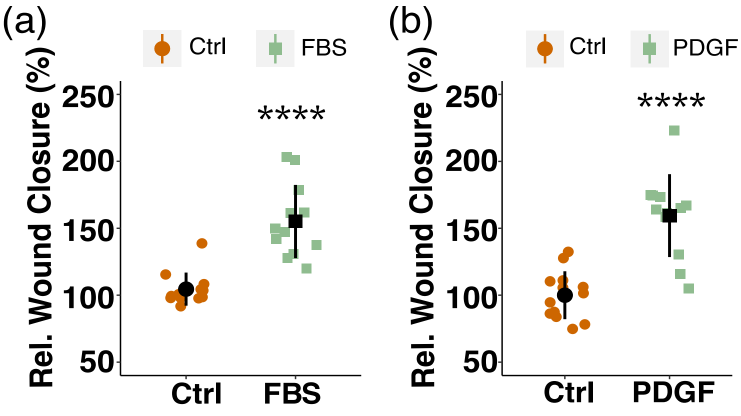

Figure S3. FBS and PDGF-BB induce migration in pericytes (PCs). PCs were grown to confluence and treated with either FBS, 2% **(a)**, PDGF-BB, 20 ng/ml **(b)** or vehicle (Ctrl) after a scratch was induced. Migration was assessed after 10h. Experiments were performed 3 times in triplicates and data represent mean +/- sd. **** $p < 0.0001$, compared to Ctrl. “Rel”: Relative.

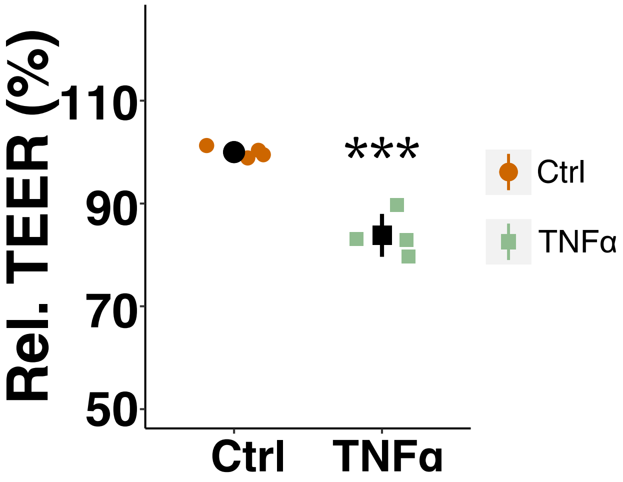

Figure S4. TNFα reduces barrier function of an endothelial monolayer in vitro. Human cerebral microvascular endothelial cells (hCMEC/D3) were cultured on transwell filters. Once a stable barrier was established after 5 days in culture, cells were treated with TNFα (10 ng/ml) or vehicle (Ctrl) and barrier function was assessed after 24h of treatment by measuring trans-endothelial electric resistance with a cellZcope instrument. Experiment was performed two times in duplicates and data represent mean +/- sd. *** $p < 0.001$, compared to Ctrl. “Rel.”: Relative.

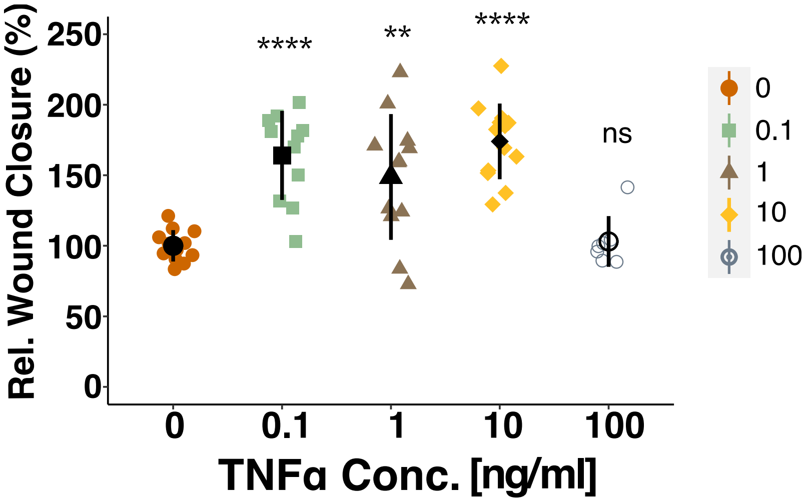

Figure S5. TNFα increases pericyte (PC) migration. Confluent PCs were scratched and treated with different concentrations of TNFα (0.1, 1, 10 and 100 ng/ml) or vehicle (0). The degree of wound closure was assessed after 10h. Experiments were performed 3 times in triplicates or quadruplicates and data represent mean +/- sd. *ns* $p > 0.05$, ** $p < 0.01$, **** $p < 0.0001$, compared to control (0). “Rel.”: Relative.

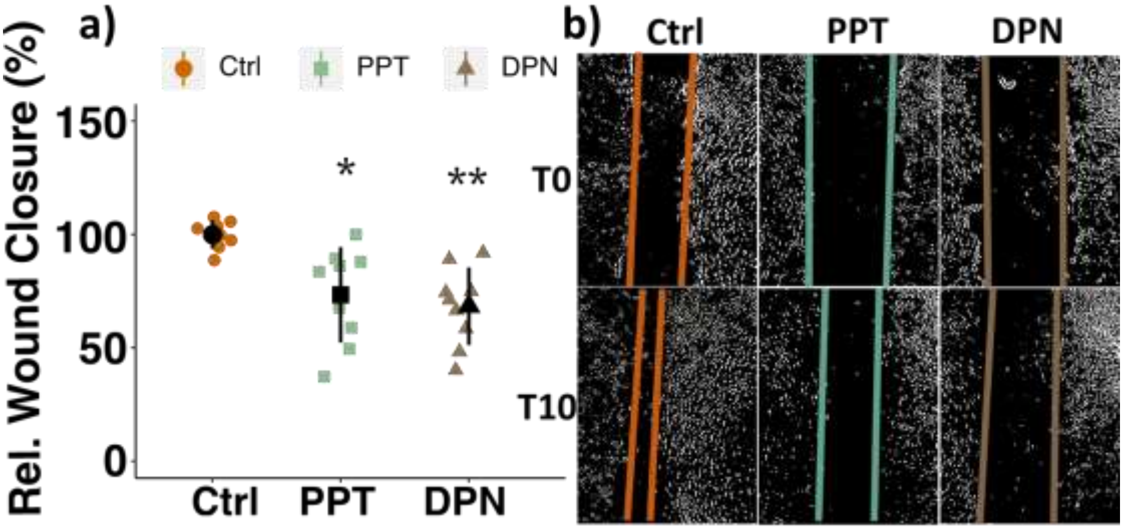

Figure S6. Estrogen receptors ER-α and ER-β are responsible for estrogen-mediated downregulation of pericyte migration. PCs were treated with agonists for ER-α (PPT) or ER-β (DPN) (10^{-7} M) or vehicle after a scratch wound was induced **(a)**. Relative wound closure was assessed after 10h and representative images are shown on the right side of the graph for T0 and T10 **(b)**. Experiments were performed 3 times in triplicates and data represent mean +/- sd. * $p < 0.05$, ** $p < 0.01$, compared to Ctrl. "Rel.": Relative.

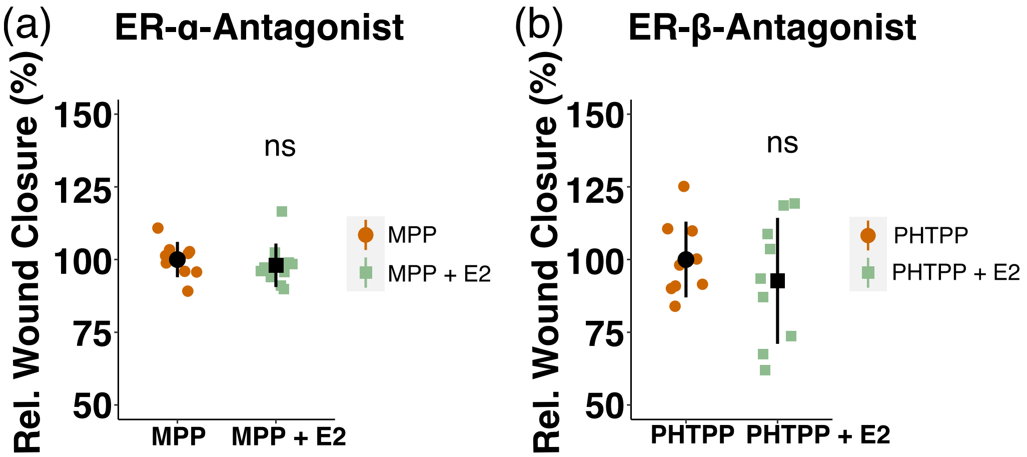

Figure S7. ER-α- and ER-β-antagonists block the effect of estradiol (E2) on pericyte (PC) migration. PCs were grown to confluency and pretreated with either one of the ER-antagonists MPP (ER-α-antagonist) **(a)** or PHTPP (ER-β-antagonist) **(b)** ($5 * 10^{-7}$ M each) for 30 minutes. Thereafter, a scratch was induced and E2 treatment (10^{-8} M) or vehicle was applied in presence of the respective antagonist. The degree of migration was assessed after 10h. Experiments were performed three times in triplicates and data represents mean +/- sd. *ns* $p > 0.05$. "Rel.": Relative.

Table S1. Top ten downregulated genes in estradiol treated pericytes.

Gene	Gene Description	Log2 FC (Co- Vs. Mono-Culture)	FDR *p*-Value
FADS1; MIR1908	Fatty acid desaturase 1; microRNA 1908	-3.1	0.208
ZFYVE16	Zinc finger, FYVE domain containing 16	-3.1	0.058
ZNF791	Zinc finger protein 791	-2.8	0.315
SIX4	SIX homeobox 4	-2.8	0.333
RBBP9	Retinoblastoma binding protein 9	-2.5	0.315
CYP1B1	Cytochrome P450, family 1, subfamily B, polypeptide 1	-2.4	0.315
CPSF2	Cleavage and polyadenylation specific factor 2	-2.3	0.208

RAB33B	RAB33B, member RAS oncogene family	-2.3	0.208
CR2	Complement component (3d/Epstein Barr virus) receptor 2	-2.3	0.305
ZDHHC20	Zinc finger, DHHC-type containing 20	-2.2	0.249

PCs were cultured on transwell inserts and treated with estradiol (10 nM) or dmso for 48h. Fold changes (FC) and adjusted *p*-values (FDR *p*-val) are depicted in the third and fourth column, respectively. Transcriptome Analysis Console (TAC, Applied Biosystems) was used for analyzing gene expression data of E2 treated vs. dmso treated PCs in triplicates. For the analysis, a fold change (FC) cut-off of +/- 2 and FDR *p*-Value of 0.34 was applied.

Table S2. Upregulated genes in estradiol treated pericytes.

**Gene**	**Gene Description**	**Log2 FC (Co- Vs. Mono-Culture)**	**FDR *p*-Value**
REXO4	REX4 homolog, 3'-5' exonuclease	2.8	0.249
NDUFAF6	NADH dehydrogenase (ubiquinone) complex I, assembly factor 6	2.4	0.249
C12orf45	Chromosome 12 open reading frame 45	2.4	0.249
VEGFC	Vascular endothelial growth factor C	2.3	0.249
ZNF582-AS1	ZNF582 antisense RNA 1 (head to head)	2.2	0.315
NPR2	Natriuretic peptide receptor 2	2.2	0.208
DACT1	Dishevelled Binding Antagonist Of Beta Catenin 1	2.1	0.332
SBSN	Suprabasin	2.1	0.249
GLIPR1N1	GLI pathogenesis-related 1 like 1	2.1	0.315

PCs were cultured on transwell inserts and treated with estradiol (10 nM) or dmso for 48h. Fold changes (FC) and adjusted *p*-values (FDR *p*-val) are depicted in the third and fourth column, respectively. Transcriptome Analysis Console (TAC, Applied Biosystems) was used for analyzing gene expression data of E2 treated vs. dmso treated PCs in triplicates. For the analysis, a fold change (FC) cut-off of +/- 2 and FDR *p*-Value of 0.34 was applied.

Table S3. Differentially regulated genes **(**DRGs) in estradiol treated endothelial cells.

**Gene**	**Gene Description**	**Log2 FC (Co- Vs. Mono-Culture)**	**FDR *p*-Value**
DLK1	Delta-like 1 homolog (Drosophila)	2.4	0.312
CYTH4	Cytohesin 4	2.3	0.171
PMAIP1	Phorbol-12-myristate-13-acetate-induced protein 1	-2.1	0.312
ING1	Inhibitor of growth family member 1	-2.1	0.321

ECs were cultured on transwell inserts before they were treated with estradiol (10 nM) or dmso for 48h. Fold changes (FC) and adjusted *p*-values (FDR *p*-val) are depicted in the third and fourth column, respectively. Transcriptome Analysis Console (TAC, Applied Biosystems) was used for analyzing gene expression data of E2 treated vs. dmso treated ECs in triplicates.. For the analysis, a fold change (FC) cut-off of +/- 2 and FDR *p*-Value of 0.34 was applied.

Table S4. Differentially regulated gene **(**DRG) in endothelial cells (ECs) co-cultured with pericytes (PCs) and treated with estradiol.

**Gene**	**Gene Description**	**Log2 FC (Co- Vs. Mono-Culture)**	**FDR *p*-Value**
ABCC3	ATP binding cassette subfamily C member 3	2.0	0.110

ECs were co-cultured with PCs on the opposite sides of transwell inserts and treated with estradiol (10 nM) or dmso for 48h. Fold changes (FC) and adjusted *p*-values (FDR *p*-val) are depicted in the third and fourth column, respectively. Transcriptome Analysis Console (TAC, Applied Biosystems) was used for analyzing gene expression data of E2 treated vs. dmso treated ECs in triplicates. For the analysis, a fold change (FC) cut-off of +/- 2 and FDR *p*-Value of 0.34 was applied.

Table S5. Downregulated genes in estradiol treated pericytes co-cultured with endothelial cells on the opposite side of a transwell insert.

Gene	Gene Description	Log2 FC (Co- Vs. Mono-Culture)	FDR *p*-Value
HIST1H3I	Histone cluster 1, H3i	-3.3	0.335
HIST1H4D	Histone cluster 1, H4d	-2.0	0.335

PCs were co-cultured with ECs on the opposite sides of transwell inserts and treated with estradiol (10 nM) or dmso for 48h. Fold changes (FC) and adjusted *p*-values (FDR *p*-val) are depicted in the third and fourth column, respectively. Transcriptome Analysis Console (TAC, Applied Biosystems) was used for analyzing gene expression data of E2 treated vs. dmso treated PCs in triplicates. For the analysis, a fold change (FC) cut-off of +/- 2 and FDR *p*-Value of 0.34 was applied.

Table S6. Top ten upregulated genes in estradiol treated pericytes co-cultured with endothelial cells on the opposite side of a transwell insert.

Gene	Gene Description	Log2 FC (Co- Vs. Mono-Culture)	FDR *p*-Value
ENPP4	Ectonucleotide pyrophosphatase/ phosphodiesterase 4 (putative)	2.7	0.335
TARSL2	Threonyl-tRNA synthetase-like 2	2.7	0.335
TIFA	TRAF-interacting protein with forkhead-associated domain	2.5	0.335
ALG6	Alpha-1,3-glucosyltransferase	2.4	0.335
MYO9A	Unconventional myosin-IXa	2.4	0.335
BEND6	BEN domain containing 6	2.3	0.335
CCDC112	Coiled-coil domain containing 112	2.3	0.335
CENPE	Centromere protein E	2.3	0.335
ZNHIT6	Zinc finger, HIT-type containing 6	2.2	0.335
PAK1IP1	PAK1 interacting protein 1	2.2	0.335

PCs were co-cultured with endothelial cells on the opposite sides of transwell inserts and treated with estradiol (10 nM) or DMSO for 48h. Fold changes (FC) and adjusted *p*-values (FDR *p*-val) are depicted in the third and fourth column, respectively. Transcriptome Analysis Console (TAC, Applied Biosystems) was used for analyzing gene expression data of E2 treated vs. dmso treated PCs in triplicates. For the analysis, a fold change (FC) cut-off of +/- 2 and FDR *p*-Value of 0.34 was applied.
